# China’s role as a global health donor in Africa: what can we learn from studying under reported resource flows?

**DOI:** 10.1186/s12992-014-0084-6

**Published:** 2014-12-30

**Authors:** Karen A Grépin, Victoria Y Fan, Gordon C Shen, Lucy Chen

**Affiliations:** Robert F. Wagner Graduate School of Public Service, New York University, 295 Lafayette Street, 3rd Floor, New York, NY USA; Center for Global Development, 2055 L St NW, Fifth Floor, Washington, DC 20037 USA; University of Hawaii at Manoa, 1960 East–West Road, Biomed D204, Honolulu, HI 96822 USA; School of Public Health, Yale University, 60 College Street, P.O. Box 208034, New Haven, CT 06520 USA; Institute for Global Health, Peking University, Beijing, China

**Keywords:** China, Africa, South-South cooperation, Development assistance for health, Foreign aid, Politics, Health systems, Malaria, Human resources for health

## Abstract

**Background:**

There is a growing recognition of China’s role as a global health donor, in particular in Africa, but there have been few systematic studies of the level, destination, trends, or composition of these development finance flows or a comparison of China’s engagement as a donor with that of more traditional global health donors.

**Methods:**

Using newly released data from AidData on China’s development finance activities in Africa, developed to track under reported resource flows, we identified 255 health, population, water, and sanitation (HPWS) projects from 2000–2012, which we descriptively analyze by activity sector, recipient country, project type, and planned activity. We compare China’s activities to projects from traditional donors using data from the OECD’s Development Assistance Committee (DAC) Creditor Reporting System.

**Results:**

Since 2000, China increased the number of HPWS projects it supported in Africa and health has increased as a development priority for China. China’s contributions are large, ranking it among the top 10 bilateral global health donors to Africa. Over 50% of the HPWS projects target infrastructure, 40% target human resource development, and the provision of equipment and drugs is also common. Malaria is an important disease priority but HIV is not. We find little evidence that China targets health aid preferentially to natural resource rich countries.

**Conclusions:**

China is an important global health donor to Africa but contrasts with traditional DAC donors through China’s focus on health system inputs and on malaria. Although better data are needed, particularly through more transparent aid data reporting across ministries and agencies, China’s approach to South-South cooperation represents an important and distinct source of financial assistance for health in Africa.

## Background

China’s contributions to global health are remarkable at a minimum because of its sheer “demographic weight” [1]. Poverty reduction and health improvements in China, which represents over one fifth of the world’s population, heavily influence global measures of disease burden as well as progress towards human development goals. However, another channel through which China also exerts influence on global health is increasingly being recognized: China’s diplomatic, political, and economic engagement on health issues with other developing countries, particularly African countries [2]. Indeed, there is growing awareness of the role of “new” vs “traditional” donors in general [3]. Such forms of “South-South Cooperation” have been met with both enthusiasm and antagonism from policymakers [4,5]. The most tangible aspect of this engagement is China’s financing of health projects in Africa. Accurate and internationally comparable data on China’s aid activities have, been hard to obtain, and so China’s role as a global health donor is less well understood and appreciated than that of more traditional global health donors [6].

There is evidence that health figures prominently among China’s foreign aid priorities. In a recent white paper released by the Information Office of China’s State Council, improving medical and health services was named as one of the key priorities on China’s foreign aid agenda [7]. While China’s engagement as a global health donor is not new – it dispatched its first medical team to Algeria in 1963 – its modalities appear to be changing and growing more prominent in Africa [2]. China hosted the first-ever Ministerial Forum on China-Africa Health Development, which was held in Beijing in August 2013 and timed to commemorate the 50th anniversary of the first Chinese medical team. At this meeting, China’s President Xi Jinping heralded a “new era” of China-Africa cooperation on health and noted that “human development is at the core of development”, which notably includes health [8].

However, China’s engagement as a global health donor in Africa is not well appreciated, which stems largely from the fact that it has been challenging to track China’s aid activities due to the fact that China employs different types of financing instruments than more traditional donors, numerous actors are involved in aid activities within China, and because China has chosen, like most emerging market countries, to not report to international aid depositories [6]. According to official statistics, China claims to have appropriated $14.41 USD billion in aid globally over 2010–12 and that just over half of that aid was allocated to Africa, but these figures have not been independently validated from other sources [7]. Recent estimates by researchers have suggested that overall annual China to Africa development financing could range from as low as $0.58 USD billion to as high as $18 USD billion [9]. This wide range is due in part to the use of different sources of data as well as different definitions of the types of development finance flows included in these estimates [9]. Additional studies have investigated the magnitude of Chinese aid to select African countries, but a lack of systematic, internationally comparable data has limited our understanding of China’s foreign aid activities [10-12]. Previous studies have also emphasized that China’s mode of engagement in Africa differs qualitatively from aid given by more traditional donors, in particular in the way in which China builds off of its own experiences as a developing country, the way in which it strikes a balance between national sovereignty and collective responsibility, and by using a mix of “hard” and “soft” instruments of national power to engender change in recipient countries [13,14].

There is a particularly poor understanding of China’s health-related activities [15]. Official estimates by the Chinese government suggest that China’s health aid in Africa doubled from US$38.5 USD million in 2006 to US$73.2 USD million in 2009, but it is unclear how these figures were generated [16]. A recent review of China’s “distinctive” engagement in global health, the most comprehensive to date, collected data on Chinese health aid activities from Chinese data sources and estimated that China provided approximately $150 USD million annually in health aid to Africa; however a more detailed breakdown of these resources, including estimates of how much individual countries were receiving in aid and for which types of activities, was not provided [17]. China claims that its priorities within the health sector include constructing health facilities, providing medicines and medical equipment, dispatching medical teams, training medical workers, and conducting exchanges and cooperation with developing countries, but these efforts have rarely been quantified or these claims verified. Qualitative case studies have validated these activities as priorities for Chinese aid, but the lack of systematic data has meant that these efforts have not been well documented or have determined the extent to which these patterns hold true in different international contexts [2,18,19]. As much of China’s health aid appears to be in kind, it is also difficult to value these projects [17]. In addition, Chinese motivations behind pledging and disbursing aid to Africa remains controversial, with some suggesting that Chinese modes of engagement are driven by economic interests and are therefore focused only on a handful of natural resource rich countries [12]. The extent to which these claims also hold true for China’s health aid has been hitherto untested.

In response to the lack of internationally comparable data on Chinese development finance flows, AidData, a partnership between the College of William and Mary, Brigham Young University, and the non-profit organization Development Gateway, developed the Tracking Under-Reported Financial Flows (TUFF) methodology to collect comprehensive and standardized data on development finance flows between China and African countries [20]. Although this data collection methodology has limitations, which are further discussed below, the AidData China to Africa Aid Database (CAAD) represents the first systematic and only publicly available database on Chinese development finance activities in Africa. In this article, we analyze the CAAD (version 1.1) to gain insights into the modes of engagement of China in global health activities in Africa from 2000–12. We triangulate the data from the CAAD to other estimates of Chinese aid and compare data on China’s global health activities in Africa to that of traditional global health donors.

## Methods

Traditional development assistance for health (DAH) databases are based on data collected by the OECD Development Assistance Committee’s (DAC) Creditor Reporting System (CRS), the most comprehensive international database of foreign aid flows [21]. Since China is not a member of the DAC and does not report to the CRS, nor does it report to other international aid repositories, directly comparable estimates of aid flows from China are not currently available. The CRS only tracks official development assistance (ODA)^a^, which is the form of development financing most commonly referred to as “aid” [21].

To address the need for more data on Chinese aid, AidData developed the TUFF methodology to collect standardized data on Chinese aid activities in Africa. AidData’s methodology is a two-stage process. In the first stage, Factiva and government websites were systematically searched for news articles, reports, and other mentions of development financing from China to individual African countries. In the second stage, trained analysts conducted additional in-depth analysis of all of the projects identified in the first stage to increase the quality of the project-level data and to generate standardized project descriptions, timelines, and estimates of the value of these projects (if available) in US dollars (USD). The second stage also includes a data quality assurance process to triangulate and check the data with additional sources. For projects with the fewest corroborating sources, additional English and Mandarin searches – using Google and Baidu, respectively – were conducted. More details of the AidData methodology can be found elsewhere [20]. The CAAD was designed to include many of the same variables contained in the CRS in order to increase the comparability of the datasets.

Although the types of development finance provided by China are not entirely comparable to that given by traditional donors [22], CAAD projects were categorized in a way to increase comparability to other international aid repositories [20]. In this study we restrict our analysis to official government financing, which in the CAAD includes projects defined as “Official Development Assistance (ODA)-like”, “Other Official Finance (OOF) Flow-like”, “Other Official Investment”, or Vague, which are projects which are either ODA-like or OOF-like but for which there is not sufficient information to distinguish between the two categories [20]. Of the 255 HPWS projects in the CAAD, 205 (or 80%) of these projects were characterized as being “ODA-like”, only 9 were characterized as OOF (3.5%), 41 were characterized as Vague (16.5%), and there were no HPWS projects that were characterized as being “Other Official Investment”. Since we are not able to discretely eliminate all OOF from the Vague projects in CAAD, and since it is challenging to identify health specific OOF from DAC, we elected to keep all data in the CAAD. This is an important limitation of our comparison of Chinese health aid with that of more traditional donors.

CAAD projects were mapped onto the standard CRS activity codes. We restricted our analysis to projects in the health (120), population (130), and water and sanitation (140) sectors. As with the CRS, HIV/AIDS-related activities are included in the population sector. In this article, we collectively refer to these three sectors as “HPWS projects.” We further restricted our analysis to all projects that were defined as firm commitments, projects that were already in the works, or completed projects. We excluded projects that had yet only been pledged from our analysis. In addition, in the CRS there is a sub-sector activity code (160.64) for social sector support to HIV/AIDS. Hence, all projects in the CAAD coded 160 were manually inspected for health-related content, yielding two additional projects that were recoded as either 120 or 130.

While there are limitations of the CAAD data collection methodology, namely biases in the types of projects that might get reported in the media, language and media-based constraints, and limits on the quantity and quality of information collected, there is significant value in analyzing this data to gain insights on China’s global health engagement in Africa. The use of datasets with similar data collection methodologies is increasingly common in many social sciences [20]. To better understand the quality of the data in the CAAD, AidData recently undertook a validation exercise of the CAAD in Uganda and South Africa. This exercise largely confirmed the value of the CAAD in practice [23]. Since many of the projects contained in the CAAD lacked a dollar amount, and due to the uncertainty in the true value of the projects, particularly those which are in kind, in the CAAD, we chose to focus most of our analysis based on the number of projects rather than the value of these projects. All dollar amounts, where available, are reported in constant 2009 USD.

To compare China to more traditional global health donors, project-level data from CRS (activity codes 120–140, plus 160.64) were also obtained for the years 2000–2012 from the OECD web portal (accessed March 1, 2014). Countries were coded using the International Organization for Standardization alpha-3 country codes.

To more qualitatively understand the types of health projects supported by China, short descriptions of each project included in the CAAD were analyzed for health and health system content. Health system keywords included words for pharmaceuticals (“drug” or “pharma”), human resources for health (“team”, “doctor”, “nurse”, “scholarship”, “midwife”, “midwive”, “personnel”, or “staff”), infrastructure (“hospital”, “construct”, or “infras”), equipment (“surg”, “equipment” or “technol”), and research (“research”). Health condition specific keywords included HIV/AIDS (“hiv” or “aids”), malaria (“malaria”), tuberculosis (“tuberculosis” or “tb”), maternal and child health conditions (“vaccine”, “immun”, “diarrhea”, “pneumonia”, “maternal”, “child”, or “measles”), and non-communicable diseases (“smoking”, “cardio”, or “cancer”).

Finally, to determine whether Chinese health aid was primarily targeted towards natural resource rich countries, we extracted data on the total nature resource rents (as a % of GDP) from the World Development Indicators [24]. We specifically used data from 2005 for all African countries.

## Results

Table 1 summarizes the main features of the CAAD. The CAAD contains detailed information on 1,686 projects that had either been committed, were being implemented, or had been completed and for the years 2000–12. Of these projects, we identified 255 HPWS projects that were allocated to 46 individual African countries or regionally. Each country received on average 5.5 HPWS projects. Health activities accounted for 79% of all HPWS projects, 5% for population activities (including HIV/AIDS), and 16% for water and sanitation projects. HPWS comprised 15.1% of CAAD projects.

**Table 1:** **Summary of projects in the China to Africa Aid Database (2000–12)**

**Health sector category**	**Number of projects**	**Percentage of all HPWS projects**	**Value of commitments (USD million)**	**Mean value of valued projects (USD million)**	**Percentage of total Chinese health aid**	**Percentage of projects with data on value**	**Percentage of projects deemed ODA like**
Health*	201	78.8	1,325	13.5	43.7	49	85.1
Population*	14	5.5	37	3.3	1.2	79	78.6
Water and Sanitation	20	15.7	1,667	57.5	55.0	73	57.5
Total HPWS projects	255		3,030				
Total CAAD projects	1,686		84,811				

*CRS activity codes are as follows: Health, 120; Population, 130; Water and Sanitation, 140. In addition, two health and population projects identified from the manual inspection of the entries coded in 160 were added to the appropriate health or population category.

As previously mentioned, not all projects in the CAAD had an assigned dollar value, and since there is great potential for discrepancies in the amounts pledged and reported in the media versus the actual amounts disbursed, the total dollar estimates need to be interpreted cautiously. Nonetheless, the CAAD contains information on 1,686 projects in 50 African countries and on $84 USD billion in commitments of official finance, of which HPWS projects represent a little more than $3 USD billion, or only 3.6% of the valued projects. There were large differences in whether an HPWS project was valued: only half of health projects had a dollar amount assigned to them in the CAAD, while 79% and 73% of population and water and sanitation projects, respectively, had been valued. Liu et al. report that health projects are more likely to be in kind, for example the deployment of medical teams, which may help to explain the lower rates of valuation in health projects [17].

Table 1 also provides the average size of valued projects within each category. Population projects tend to be the smallest (an average of $3.3 USD million per project) and unsurprisingly water and sanitation projects are the largest at $57.5 USD million per project. To try to account for unvalued projects, and simply as a thought exercise, if we naively assume that projects that were not valued were similar to those that did receive a valuation, and multiply the total number of projects in each category by the average value of valued projects in each category, we estimate that the total amount of Chinese health aid for the entire time period could be as high as $5 USD billion (calculation not shown) from 2000–12, however, we note this figure is highly speculative and do not place much weight on this estimate.

**Figure 1:**
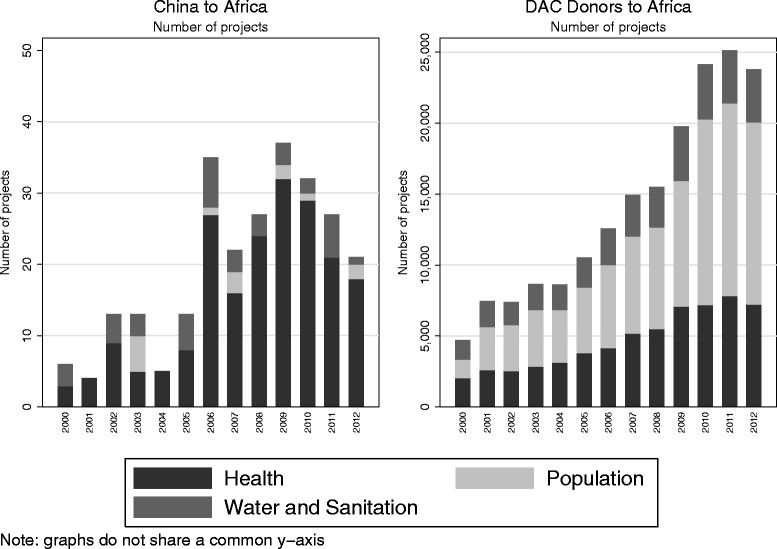
**Number of projects by donor, sector, and by year (2000–12).**

Figure 1 compares the trends in number of projects from China relative to DAC donors by year and by sector. A number of interesting observations can be made. First, similar to DAC donors, China increased the number of projects it supported in Africa over 2000–09 but appears to have slightly decreased the number of projects thereafter. Second, China appears to have different health priorities than DAC donors: nearly half (48%) of all HPWS projects by DAC donors to African countries over 2000–12 supported population programs, mostly HIV/AIDS projects [25]. By contrast, only 5% of HPWS Chinese projects supported population programs. In fact, further analysis of the descriptions of these projects revealed that only two HPWS projects in the CAAD included the words “HIV” or “AIDS”. Most of the increases in the number of Chinese projects were driven by increases in the number of health projects, whereas the increase in projects from DAC donors was primarily driven by the increase in population projects.

**Figure 2:**
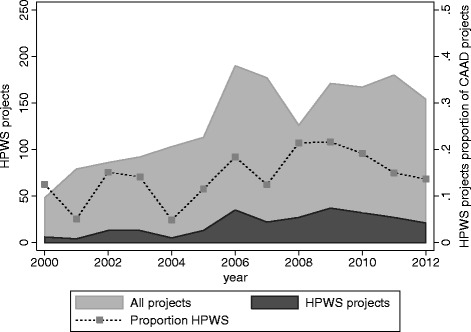
**Health, population, water, and sanitation projects as a share of all China to Africa aid projects (2000–12).**

Figure 2 investigates whether the increase in number of HPWS projects reflects a general trend in all development projects from China to Africa, or whether health has increased as a priority on China’s foreign aid agenda. Health appears to have increased as a priority through 2009; however, in recent years health projects as proportion of all projects has decreased slightly.

Table 2 compares the magnitude of Chinese health aid to Africa relative to other bilateral DAC donors, by comparing the total amount of US$ committed bilaterally (which excludes their donations to multilateral institutions) by the top 10 donors. Limitations of the data notwithstanding, China would rank ninth in bilateral health aid to Africa among bilateral donors, behind the Netherlands but ahead of Denmark. Since many of the other top ranked bilateral donor countries are also major multilateral donors, and China has given relatively little to global health multilateral programs until more recently, this ranking should be interpreted *only* with regards to bilateral contributions [17,26].

**Table 2:** **Bilateral health aid contributions to Africa, by donor (2000–12)**

**Rank**	**Donor country**	**Commitments to HPWS projects (USD million)**	**Average annual contribution (USD million)**
1	United States	31,626.5	2,432.8
2	EU Institutions	8,796.8	676.7
3	United Kingdom	6,050.5	465.4
4	France	5,001.9	384.8
5	Germany	4,949.7	380.7
6	Japan	4,456.3	342.8
7	Canada	4,065.4	312.7
8	Netherlands	3,903.7	300.3
9	**China**	**3,029.6**	**233.0**
10	Denmark	2,200.1	169.2

Source: CAAD (China, in bold) and OECD CRS (other donors). Figures refer to only bilateral health aid contributions.

Table 3 provides a ranking of the top 10 recipients of health aid from both Chinese and DAC donors. Only two countries (Ghana and Kenya) make it onto both top 10 lists, suggesting low levels of overlap China and more traditional donors in terms of where they target their aid. Chinese aid also appears to be much more heavily concentrated than DAC donors: their top 10 recipients received 89% of all Chinese health aid whereas the top 10 recipients of aid from DAC donors only captured 55% of DAC donor commitments.

**Table 3:** **Highest Total Commitments to HPWS by African Country (2000–12)**

**African Country**	**HPWS From China (USD million)**	**African Country**	**HPWS From DAC Donors (USD million)**
Cameroon	840.9	Nigeria	10,502.6
Ghana	740.1	Ethiopia	8,981.2
Kenya	271.1	Tanzania	8,818.0
Sudan	227.7	Kenya	8,654.9
Zimbabwe	223.7	Uganda	6,54.9
Angola	107.8	Mozambique	6,295.1
Mauritius	84.9	South Africa	6,060.6
Côte d’Ivoire	80.6	D.R. Congo	5,237.8
Zambia	64.7	Zambia	4,814.9
Niger	64.7	Ghana	4,335.9
Sum of top 10 recipients	2,706.1		70,248.0

Source: CAAD for China and OECD CRS for other DAC donors. Figures refer to only bilateral contributions and, in the case of China, only to valued projects.

**Figure 3:**
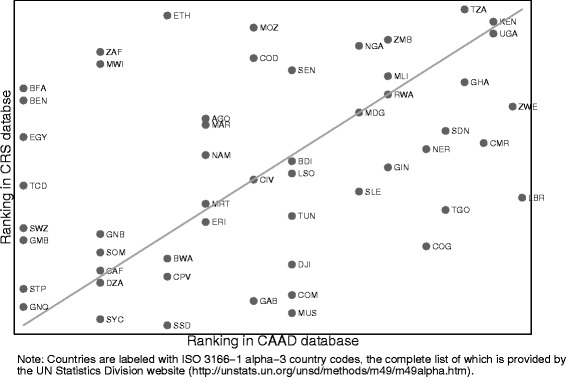
**Prioritization of recipient countries by China and by other bilateral donors.**

Figure 3 illustrates the relative priority given to individual recipient countries by China versus DAC donors. Each recipient country is ranked on the number of projects it received from China (*x*-axis, increasing priority from left to right) and DAC donors (*y*-axis, increasing priority from bottom to top). The diagonal line represents the point of equal prioritization. There appears to be moderate correlation between the priority given to African countries by China and DAC donors (Spearman correlation coefficient of 0.44). However, there are a few notable outliers, including a number of countries that received no funding from China, namely Egypt (EGY), Benin (BEN), the Gambia (GMB), Equatorial Guinea (GNQ), Burkina Faso (BFA), Chad (TCD), Swaziland (SWZ), and Sao Tome and Principe (STP). Interestingly, the Gambia, Burkina Faso, Sao Tome and Principe, and Swaziland have all recognized the sovereignty of Taiwan, which appears to have made them ineligible for Chinese support. Although it has been suggested that China is using its foreign aid as a means to secure natural resources and expand its commercial market, it is not obvious from our data that this is in fact the case for health [12]. While some natural-resource rich countries such as Sudan (SDN), Ghana (GHA), and Niger (NER) appear to have more priority from China, there are also examples of other naturel resource rich countries that receive less priority from China including Zambia (ZMB), Nigeria (NGA), and the Democratic Republic of the Congo (COD). Resource rich countries actually appear to be prioritized by both types of donors.

**Figure 4:**
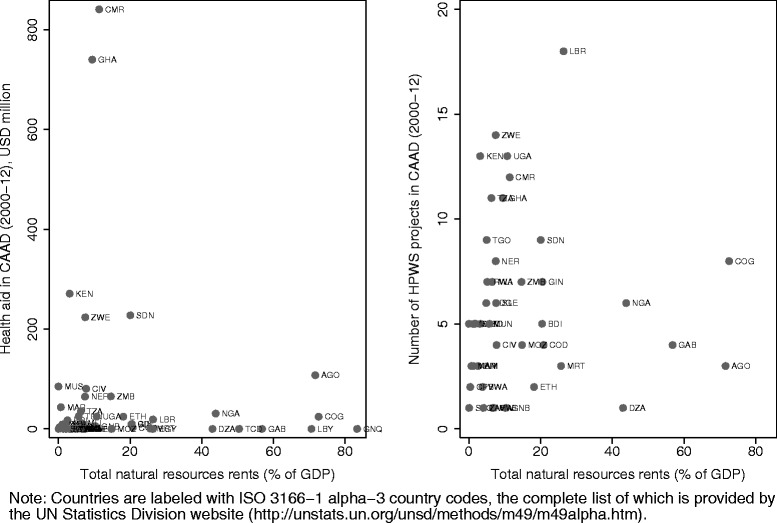
**Relationship between Chinese aid and natural resources.**

To more formally test whether or not natural resources are an predictor of health aid from China, we plot both the total amount of funding each African country received in Chinese health aid over 2000–12 and the number of projects it received from China relative to the proportion of its GDP that represent natural resource rents, which include oil rents, natural gas rents, coal rents, mineral rents, and forest rents (using data from 2005 for all countries). These data are plotted in Figure 4. There does not appear to be any significant relationship between either of these indicators of Chinese aid with resource rents. If we crudely regress measures of Chinese aid (number of projects and total funding) on this measure of natural resource rents, the coefficients on rents are actually negative but not statistically significant, suggesting that there is no positive relationship between these indicators for Chinese health aid as has been suggested by observers for Chinese aid in general (not shown).

**Figure 5:**
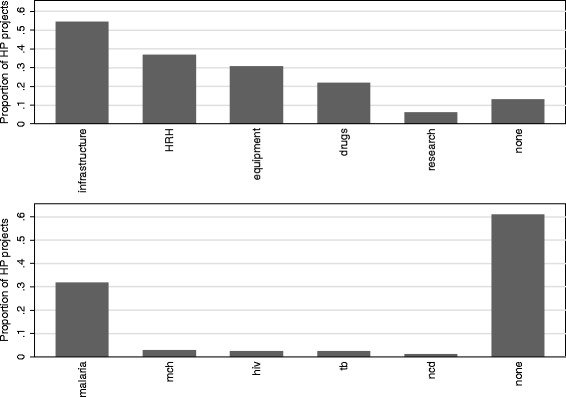
**Content analysis of Chinese aid projects by health system and disease-specific keywords.**

Figure 5 summarizes the analysis of health keywords in the project descriptions. We exclude water and sanitation projects from this analysis since most of these projects support infrastructure and are by nature not targeted to health systems or to specific disease conditions. Projects can independently be tagged with more than one keyword and hence sum to more than 100%. More than 50% of the health and population (HP) projects in the sample target infrastructure. This includes many large hospital construction or renovation projects.

Human resources for health (HRH) make up a significant (40%) portion of the health aid portfolio. Chinese aid in HRH is provided as either deployment of medical teams or training of local health personnel. Chinese medical teams make up a major part of the HRH portfolio: Liu et al. [17] appraised the value of Chinese medical teams in Africa to be about $60 USD million annually from 2007 to 2011. Training of health personnel can include training that happens in Africa or in China: China provides scholarships for foreign medical students to be trained in Chinese universities and also supports government officials, technical professionals, and students to participate in training and education taking place in African countries.

The provision of equipment (a quarter of HP projects) and drugs (a fifth of HP projects) is also common. Among the health conditions targeted by Chinese aid, only malaria appears to a priority for the China, with about a third of projects targeting malaria. Many of these malaria projects were also tagged for pharmaceuticals, suggesting that donations and distribution of anti-malaria drugs (i.e. artemisinin), as previous studies have suggested, are in fact common [27,28].

## Discussion

This article provides a multi-country description of Chinese official health financing for health related activities in Africa. Using AidData’s CAAD, we identified 255 HPWS projects from China to 46 African countries, with an estimated value of approximately $3 USD billion in financing over 2000–12. Over time, health financing from China to Africa has increased in terms of both the number of projects and as a proportion of all Chinese development finance flows to the continent. Chinese health developing financing is large and would rank it among the leading bilateral global health donors to Africa. China, however, appears to have very different health priorities than DAC donors, with much more emphasis on health system projects and HRH, and much less emphasis on disease-specific programs, notably HIV/AIDS, which has been the predominant priority of DAC donors in Africa [25]. We do not find evidence that Chinese health aid is targeted specifically to resource rich countries. Rather, we find most countries in Africa have received some aid from China during the period of study.

Despite the important caveats mentioned above in the valuation of projects, our estimates suggest that China has been pledging health projects valued at $231 USD million a year on average to 46 African countries over the past decade or roughly $5 USD million per country per year. However, Chinese aid appears to be more concentrated than that of more traditional donors: the top 10 recipient countries received 89% of Chinese health aid to Africa.

By comparison, using 529 sources of manually collected information, Liu et al. [17] estimated China disbursed $150 USD million in health aid annually, but likely excluded projects in water and sanitation. Given these differences as well as the differences in methods employed in collecting data, we believe that these two estimates corroborate each other. Our estimate of China’s health aid is however, higher than figures released by the Chinese government in 2011 [7]. Chinese officials themselves have lamented about the challenges in tracking their own aid flows [29]. While the total amount of money we identify, roughly $3 USD billion in funding over 12 years, is relatively large, spread out among the large number of countries in Africa receiving support from China, this would not translate into large amounts of aid per country, which might also help to explain why much of China’s aid activities have received little attention from the global health policy community.

There are many limitations in the data employed in this study. The CAAD should be interpreted with caution given its different methodological approach to data collection than the CRS. In some cases, the CAAD may overestimate the value of aid projects, as the media may be more likely to report larger rather than smaller projects. Plus, the media may report a project at the announcement stage, at which point funds are only committed and not the actual amount of the funds that are disbursed. The CAAD also cannot identify projects that are canceled before implementation. The CAAD may also underestimate the true value of China’s development finance flows since not all projects were valued and the use of triangulating news reports to government reports accessible to the public is only a proxy for official government reporting. The mere fact that fewer than half of the health projects and only three quarters of the population and water and sanitation projects were valued in the CAAD suggests that valuation of projects is not even across categories, adding to the difficulty of extrapolating the value of non-valued projects. Compared to water and sanitation projects, health projects are more likely to be in kind [17] – through the exchange of visiting health workers and the transfer of medicines, equipment, and other supplies, which could make it more difficult to appraise the value of these projects. In addition, the CAAD data cannot be seen as representative of all of China’s development finance activities, in particular, we focus mainly on projects that are predominantly ODA-like, but we recognize differences remain between these datasets.

For these reasons, this paper has emphasized the descriptive uses of the CAAD data. Our study findings largely corroborate with what others have identified in more qualitative analysis or in country case studies, with some notable differences, in particular the claims of emphasis on HIV/AIDS projects and the targeting of resources to natural resource rich countries.

Despite the difficulty of valuing in-kind projects, the costs per unit of health service delivered financed by China’s national and provincial governments is likely lower compared to traditional western donors [30]. For example, the cost of paying the salary of a doctor originating from China and supported by his or her given Chinese provincial government dispatched to a given African country as part of a Chinese medical team is certainly lower than the cost of paying an international NGO’s doctor salary supported by a Western Government. As a rough back-of-the-envelope calculation, 1160 Chinese medical workers were deployed in 2013 and were valued at $60 USD million annually by Liu et al. [17], or about $50,000 per worker (which includes not only salary, fringe benefits, housing, food, and travel costs, but also the project’s accompanying medicines and medical equipment as well).

## Conclusions

African countries require additional funding to meet the Millennium Development Goals (soon the Sustainable Development Goals), achieve Universal Health Coverage, and other global health priorities, and given that aid flows from traditional donors have plateaued in recent years, there is a need to find new sources of funding for these additional resources [31]. This study suggests that China may already be helping to fill these needs by funding health activities in ways that are complimentary to those provided by DAC donors. Plus, China’s engagement with its African partners is qualitatively different and, according to the government policies, guided by principles of non-interference with domestic affairs of a recipient country, mutual benefits in economic development, and self-determination among partner countries, which continues to be enshrined in its government policies.

Based on its recent experience with economic development and improved population health, China may have more to offer African countries than just financial resources. The challenges China historically faced in raising population health status were embedded in the health sector and related sectors, such as agriculture, education, and the environment. The same holds true for African countries today. China has acknowledged these parallels when charting out a long-term vision and financial commitment to partner countries during high-level policy dialogues. Chinese policy-makers also believe that such shared experiences deepens their diplomatic relationships with Sub-Saharan African countries.

China’s engagement in global health is rapidly expanding in other ways beyond the financing of projects in other countries [32-34]. It has, for example, been a leading player in promoting and supporting inter-BRICS country initiatives in health [33]. Public-private partnerships and institutional capacity-building for R&D between China and other low- and middle-income countries have led to new, lower-cost product pipelines for new drugs, vaccines, and diagnostics [35]. China has begun to play an increasingly strategic role in providing technical assistance to missions related to infectious disease control [36]. This engagement has been noted in the media coverage of the current outbreak of Ebola in West Africa, where China is among the leading donors to the development response and quickly sent a large number of medical teams to the affected countries. To date China’s contributions to multilateral programs has been minimal, but there are signs it may be increasing its commitments to such efforts [32].

Given China’s recent experiences in health system reform and signaled shift at the recent Beijing Declaration towards “universal coverage of health services” and “sustainable, long-term health solutions”, China’s government has an opportunity and a potentially comparative advantage over other bilateral donors to provide different types of support to African countries [37-39]. Nevertheless, all donors including China must consider improving their development activities to support increased aid effectiveness, which could include the use of performance-based financing and improved performance verification, the use of objective criteria to make allocations across countries, and greater experimentation and learning [40-43]. Imposing health priorities on other countries may have unintended consequences on African’s relatively weak health systems [25,44]. At the very least, an important first step will be to increase the transparency around its health and development related activities in other countries so that recipient countries policy makers alike are better able to track and measure the impact of China’s official health financing activities in Africa [6].

### Endnote

^a^The DAC defines ODA as “grants or loans to countries and territories and to multilateral agencies which are: (a) undertaken by the official sector; (b) with promotion of economic development and welfare as the main objective; (c) at concessional financial terms (if a loan, having a grant element of at least 25 per cent).

## References

[1] Han Q, Chen L, Evans T, Horton R (2008). China and global health. The Lancet.

[2] Youde J (2010). China’s Health Diplomacy in Africa. China Int J.

[3] Dreher A, Fuchs A, Nunnenkamp P (2013). New Donors, International Interactions. Empirical and Theoretical Research in International Relations.

[4] **China-Africa: A Partnership For Global Health That Puts People First.** [http://onlinedigeditions.com/article/China-Africa%3A + A + Partnership + For + Global + Health + That + Puts + People + First/1526661/0/article.html]

[5] **Hillary Clinton launches African tour with veiled attack on China | World news | theguardian.com.** [http://www.theguardian.com/world/2012/aug/01/hillary-clinton-africa-china]

[6] Fan VY, Grépin KA, Shen GC, Chen L (2014). Tracking the flow of health aid from BRICS countries. Bull WHO.

[7] **White Paper on China’s Foreign Aid [English Translation].** [http://news.xinhuanet.com/english/china/2014-07/10/c_133474011.htm]

[8] **China to strengthen cooperation with Africa on health.** [http://wcm.fmprc.gov.cn/pub/zflt/eng/zfgx/zzjw/t1067444.htm]

[9] **China’s Development Finance to Africa: A Media-Based Approach to Data Collection.** [http://www.cgdev.org/publication/chinas-development-finance-africa-media-based-approach-data-collection]

[10] **U.S. GAO - Sub-Saharan Africa: Trends in U.S. and Chinese Economic Engagemen.** [http://www.gao.gov/products/GAO-13-199]

[11] Tan-Mullins M, Mohan G, Power M (2010). Redefining “Aid” in the China-Africa Context. Dev Change.

[12] Bräutigam D (2009). The Dragon’s Gift: the Real Story of China in Africa.

[13] King K (2013). China's Aid and Soft Power in Africa: The Case of Education and Training.

[14] Large D (2007). Beyond “Dragon in the Bush”: The Study of China Africa Relations. African Affairs.

[15] **China’s Emerging Global Healthand Foreign Aid Engagement in Africa.** [http://csis.org/files/publication/111122_Freeman_ChinaEmergingGlobalHealth_Web.pdf]

[16] **Implementation of the Follow-up Actions of the Beijing Summit of the Forum on China-Africa Cooperation.** [http://www.focac.org/eng/dsjbzjhy/hywj/t627504.htm]

[17] Liu P, Guo Y, Qian X, Tang S, Li Z, Chen L (2014). China’s distinctive engagement in global health. The Lancet.

[18] Li A (2011). Chinese Medical Cooperation in Africa.

[19] Huang Y (2010). Pursuing health as foreign policy: the case of China. Indiana J Global Legal Studies.

[20] Strange A, Parks BC, Tierney MJ, Fuchs A, Dreher A, Ramachandran V: **China’s Development Finance to Africa: A Media-Based Approach to Data Collection.** In *CGD Working Paper 323.* Washington DC: Center for Global Development; 2013.

[21] Grépin KA, Leach-Kemon K, Schneider M, Sridhar D (2012). How to do (or not to do) … Tracking data on development assistance for health. Health Policy Plan.

[22] Bräutigam D (2011). Aid “With Chinese Characteristics”: Chinese Foreign Aid and Development Finance Meet the OECD‐DAC Aid Regime. J Int Dev.

[23] Muchapondwa E, Nielson D, Parks B, Strange AM: **“Ground-truthing” Chinese development finance in Africa**. 2014. ISSN 1798-7237 ISBN 978-92-9230-752-3

[24] **Total natural resources rents (% of GDP) | Data.** [http://data.worldbank.org/indicator/NY.GDP.TOTL.RT.ZS]

[25] Grépin KA (2012). HIV donor funding has both boosted and curbed the delivery of different non-HIV health services in sub-Saharan Africa. Health Affairs.

[26] Sridhar D, Brolan CE, Durrani S, Edge J, Gostin LO, Hill P, McKee M (2013). Recent Shifts in Global Governance: Implications for the Response to Non-communicable Diseases. PLoS Med.

[27] Weiyuan C (2009). Ancient Chinese anti-fever cure becomes panacea for malaria. Bull WHO.

[28] Siringi S (2003). Africa and China join forces to combat malaria. The Lancet.

[29] 中华人民共和国国家卫生和 计划生育委员会: 中非部长级卫生合作发展会议北京宣言 [http://www.moh.gov.cn/lbwz/ptpjj/201308/d331f487fa304e11b86a856df7f4eee8.shtml]

[30] Shen GC, Fan VY (2014). China’s provincial diplomacy to Africa: applications to health cooperation. Contemporary Politics.

[31] Murray CJL, Anderson B, Burstein R, Leach-Kemon K, Schneider M, Tardif A, Zhang R (2011). Development assistance for health: trends and prospects. Lancet.

[32] Minghui R, Guoping L (2014). China’s global health strategy. The Lancet.

[33] da Silva JB, Desiraju K, Matsoso P, Minghui R, Salagay O (2014). BRICS cooperation in strategic health projects. Bull WHO.

[34] McKee M, Marten R, Balabanova D, Watt N, Huang Y, Finch A, Fan VY, Van Damme W, Tediosi F, Missoni E (2014). BRICS’ role in global health and the promotion of universal health coverage: the debate continues. Bull WHO.

[35] Miloud Kaddar JMSS (2014). Impact of BRICS’ investment in vaccine development on the global vaccine market. Bull WHO.

[36] **China and global health.** [http://www.unspecial.org/wp-content/uploads/2014/05/UNSpecial_Mai2014.pdf]

[37] 中非部长级卫生合作发展会议北京宣言 - 中华人民共和国国家卫生和计划生育委员会 [http://www.chinapop.gov.cn/gjhzs/s3590/201308/da8ad62e487a481f987e631e1318c6fc.shtml]

[38] Open Society Foundation for South Africa: **Beijing Declaration of the Ministerial Forum of China–Africa Health Development.** In *ᅟ.* Pineland: South African Foreign Policy Initiative 2014; ᅟ [http://www.safpi.org/news/article/2013/beijing-declaration-ministerial-forum-china-africa-health-development]

[39] Yip WC-M, Hsiao WC, Chen W, Hu S, Ma J, Maynard A (2012). Early appraisal of China’s huge and complex health-care reforms. The Lancet.

[40] Fan VY, Duran D, Silverman R, Glassman A (2013). Performance-based financing at the Global Fund: an analysis of grant ratings and funding, 2003–12. Lancet Global Health.

[41] Sridhar D (2010). Seven challenges in international development assistance for health and ways forward. J Law Med Ethics.

[42] Fan VY, Glassman A, Silverman R: **How a new funding model with shift allocations from the Global Fund to Fight AIDS, Tuberculosis, and Malaria.** *Health Affairs*, 2014 Nov 12 10.1377/hlthaff.2014.0240 25392001

[43] Bump J, Fan VY, Lanthorn HE, Yavuz EN (2012). In the Global Fund's court: experimentation, evaluation, and the AMFm. Lancet.

[44] Fan VY, Silverman R, Glassman A (2012). Does HIV/AIDS funding undermine health systems?. Am J Tropic Med Hygiene.

